# Addressing Diabetes and Poorly Controlled Hypertension: Pragmatic mHealth Self-Management Intervention

**DOI:** 10.2196/12541

**Published:** 2019-04-09

**Authors:** Allison A Lewinski, Uptal D Patel, Clarissa J Diamantidis, Megan Oakes, Khaula Baloch, Matthew J Crowley, Jonathan Wilson, Jane Pendergast, Holly Biola, L Ebony Boulware, Hayden B Bosworth

**Affiliations:** 1 Durham Center of Innovation to Accelerate Discovery and Practice Transformation Durham Veterans Affairs Health Care System Durham, NC United States; 2 Division of Nephrology Department of Medicine Duke University School of Medicine Durham, NC United States; 3 Duke Clinical Research Institute Duke University School of Medicine Durham, NC United States; 4 Gilead Sciences, Inc Foster City, CA United States; 5 Division of General Internal Medicine Department of Medicine Duke University School of Medicine Durham, NC United States; 6 Department of Population Health Sciences Duke University School of Medicine Durham, NC United States; 7 Outcomes Research Group Duke Clinical Research Institute Duke University School of Medicine Durham, NC United States; 8 Division of Endocrinology, Diabetes and Metabolism Department of Medicine Duke University School of Medicine Durham, NC United States; 9 Department of Biostatistics and Bioinformatics Duke University School of Medicine Durham, NC United States; 10 Department of Family Medicine Lincoln Community Health Center Durham, NC United States; 11 Department of Psychiatry and Behavioral Sciences Duke University School of Medicine Durham, NC United States; 12 School of Nursing Duke University Durham, NC United States

**Keywords:** telemedicine, cardiovascular diseases, diabetes mellitus type 2, vulnerable populations, renal insufficiency, professional-patient relations, hypertension

## Abstract

**Background:**

Patients with diabetes and poorly controlled hypertension are at increased risk for adverse renal and cardiovascular outcomes. Identifying these patients early and addressing modifiable risk factors is central to delaying renal complications such as diabetic kidney disease. Mobile health (mHealth), a relatively inexpensive and easily scalable technology, can facilitate patient-centered care and promote engagement in self-management, particularly for patients of lower socioeconomic status. Thus, mHealth may be a cost-effective way to deliver self-management education and support.

**Objective:**

This feasibility study aimed to build a population management program by identifying patients with diabetes and poorly controlled hypertension who were at risk for adverse renal outcomes and evaluate a multifactorial intervention to address medication self-management. We recruited patients from a federally qualified health center (FQHC) in an underserved, diverse county in the southeastern United States.

**Methods:**

Patients were identified via electronic health record. Inclusion criteria were age between 18 and 75 years, diagnosis of type 2 diabetes, poorly controlled hypertension over the last 12 months (mean clinic systolic blood pressure [SBP] ≥140 mm Hg and/or diastolic blood pressure [DBP] ≥90 mm Hg), access to a mobile phone, and ability to receive text messages and emails. The intervention consisted of monthly telephone calls for 6 months by a case manager and weekly, one-way informational text messages. Engagement was defined as the number of phone calls completed during the intervention; individuals who completed 4 or more calls were considered engaged. The primary outcome was change in SBP at the conclusion of the intervention.

**Results:**

Of the 141 patients enrolled, 84.0% (118/141) of patients completed 1 or more phone calls and had follow-up SBP measurements for analysis. These patients were on average 56.9 years of age, predominately female (73/118, 61.9%), and nonwhite by self-report (103/118, 87.3%). The proportion of participants with poor baseline SBP control (50/118, 42.4%) did not change significantly at study completion (53/118, 44.9%) (*P*=.64). Participants who completed 4 or more phone calls (98/118, 83.1%) did not experience a statistically significant decrease in SBP when compared to those who completed fewer calls.

**Conclusion:**

We did not reduce uncontrolled hypertension even among the more highly engaged. However, 83% of a predominately minority and low-income population completed at least 67% of the multimodal mHealth intervention. Findings suggest that combining an automated electronic health record system to identify at-risk patients with a tailored mHealth protocol can provide education to this population. While this intervention was insufficient to effect behavioral change resulting in better hypertension control, it does suggest that this FQHC population will engage in low-cost population health applications with a potentially promising impact.

**Trial Registration:**

ClinicalTrials.gov NCT02418091; https://clinicaltrials.gov/ct2/show/NCT02418091 (Archived by WebCite at http://www.webcitation.org/76RBvacVU)

## Introduction

Diabetes and hypertension are two of the most prevalent chronic illnesses worldwide. Patients with comorbid diabetes and hypertension are at greater risk for progressive renal and cardiovascular complications, including diabetic kidney disease (DKD) [1-6]. Improving long-term outcomes in this high-risk group depends on identifying patients with diabetes and poorly controlled hypertension, addressing modifiable risk factors, and ensuring access to optimal treatment and education early in the patient’s disease trajectory [4,7].

The incidence and prevalence of diabetes and hypertension continue to disproportionately impact individuals who are minorities and/or of lower socioeconomic status populations [7-9]. Reasons for higher rates of diabetes and hypertension in these populations can include limited access to quality medical care, low health literacy, and lack of insurance [7,10,11]. These challenges and barriers to engaging in self-management place patients of lower socioeconomic status populations at risk for poorer health and adverse renal and cardiovascular outcomes [12]. Identifying methods to deliver personalized, disease-specific support may help decrease the potential impact of diabetes and poorly controlled hypertension.

Patients with diabetes and poorly controlled hypertension benefit from aggressive treatment that improves control of modifiable risk factors such as management of blood glucose, blood pressure, diet, exercise, and weight and smoking cessation [13-15]. Simultaneously addressing these factors may preserve renal function and delay a decline in renal function and death [7-9]. Patient-centered interventions that address multiple factors and facilitate personalized problem solving may be more effective than interventions with a generalized approach [3].

Mobile health (mHealth) is one way to provide patient-centered information and promote engagement in self-management for patients who are at high risk for DKD. Mobile health interventions are effective ways to change behaviors in individuals with chronic illnesses such as diabetes and hypertension [16-18]. Interventions via mHealth have high potential reach, as a large percentage of US adults have a mobile phone [19], including individuals with low socioeconomic status [20-22]. Additionally, mHealth technologies are inexpensive and can be easily scaled, thus increasing the potential for behavioral intervention dissemination. However, there has been a lack of studies using mHealth to reduce risk factors among ethnic and racial minorities and low-income individuals who are at particularly high risk for poor renal and cardiovascular outcomes [23,24].

The purpose of this feasibility study was to pilot test an mHealth intervention among individuals with diabetes and poorly controlled hypertension. The intervention included tailored behavioral-educational components with a focus on disease self-management.

## Methods

### Study Design

This was a single-arm, pragmatic study designed to implement a 6-month intervention. This multifactorial intervention simultaneously addressed multiple risk factors for adverse renal outcomes through a combination of patient self-monitoring, behavioral therapies, and education that optimized adherence and improvements in health behavior self-efficacy. As a proxy for improvements in these process measures, we examined whether the intervention was associated with a positive impact on systolic blood pressure. Duke University’s institutional review board approved this study (Pro00052081), and this study was registered with ClinicalTrials.gov (NCT02418091).

### Setting

We recruited patients from a federally qualified health center (FQHC) in a midsize city in the southeastern United States. We chose this FQHC due to the high incidence of poorly controlled diabetes and hypertension in this underserved population. In 2017, this FQHC provided comprehensive primary and preventive care to over 33,500 unique individuals. This FQHC serves a population that is predominantly Latinx (47%) or black (39%), and many patients in this clinic system (50% of adults and 24% of children) are uninsured. Overall, patients seeking care in this clinic are lower income: 38% of patient households make less than 100% of the federal poverty level and only 19% make above 100% of the federal poverty level (43% did not provided income documentation).

### Recruitment and Enrollment

We identified eligible patients using the FQHC’s electronic health record (EHR). Inclusion criteria for this study included age between 18 and 75 years, diagnosis of type 2 diabetes (International Classification of Disease [ICD]-9 codes 250.x0, 250.x2; ICD-10 codes E11.0-E11.9), poorly controlled hypertension (1-year mean clinic systolic blood pressure [SBP] ≥140 mm Hg and/or diastolic blood pressure [DBP] ≥90 mm Hg), and access to a mobile phone and ability to receive text messages and emails. Exclusion criteria included inability to speak English, residence in a nursing home or long-term care facility or receipt of home health care, or a current diagnosis of pancreatic insufficiency or diabetes secondary to pancreatitis. We excluded patients who self-reported alcohol use of more than 14 alcoholic beverages per week because this study was not designed to address substance abuse behaviors.

We identified patients using the FQHC’s EHR and then screened and identified eligible patients. Eligible patients received a letter from their primary care provider requesting study participation. After a 10-day period in which potential patients could opt out of the study, patients were contacted by the call center affiliated with the local academic clinical research organization. Patients were informed and consented over the phone using an institutional review board–approved script. All patients who verbally consented to the study were enrolled.

### Simultaneous Risk Factor Control Using Telehealth to Slow Progression of Diabetic Kidney Disease Automated Population Program

The Simultaneous Risk Factor Control Using Telehealth to Slow Progression of Diabetic Kidney Disease Automated Population Program (STOP-DKD APP) consisted of two novel platforms. The first platform was an electronic registry that used the clinic’s EHR to identify the target population (eg, patients with diabetes and poorly controlled hypertension). This was combined with a second electronic platform that delivered an evidence-based behavioral intervention to improve self-management comprising educational content on diabetes and hypertension [25]. The goal of the intervention was not to replace clinic-based management but to supplement it in order to more efficiently intensify therapy that may otherwise be left until future appointments. Information for all eligible patients was entered into the STOP-DKD APP platform. The STOP-DKD APP intervention was guided by three behavioral science models: chronic care model [26,27], health decision model [28], and the transtheoretical model [29]. These complementary models informed the design of the intervention to slow DKD progression in the population.

The chronic care model describes factors that can improve functional and clinical outcomes, particularly in chronic conditions [26,27]. Patients with DKD and uncontrolled hypertension often receive suboptimal care due to fragmented and poorly designed health systems. Thus, the model acknowledges that a substantial portion of chronic care takes place outside of formal health care settings and highlights six core elements for the provision of optimal care of patients with chronic disease. The STOP-DKD APP intervention addressed the core elements described in the chronic care model [26,27] in order to create a more proactive provider team and more engaged patient by (1) engaging health systems interested in improvement strategies, (2) leveraging an innovative clinical information system to identify study patients and community resources to tailor information and feedback, (3) using decision support informed by health behavior models to optimize patient self-management, and (4) redesigning the delivery system by using case managers to facilitate management of complex medication regimens in close communication with participant primary care providers [30-32]. These intervention strategies created a more proactive provider team and more active patient.

We used the health decision model to guide the selection of behaviors related to treatment adherence, diabetes, and poorly controlled hypertension [28]. For patients with DKD and poorly controlled hypertension, the complexity of care requires that appropriate behavior change theories be applied toward understanding behaviors related to treatment adherence. To focus on health decisions, the health decision model draws upon other behavioral models to combine the influences of health beliefs and modifying factors with contributions from the patient preference literature, including important factors such as memory and the experience of side effects associated with medications [28,29,33,34]. The health decision model also identifies potential behavioral factors that may explain poor disease control related to treatment adherence by examining factors that hinder or promote health behaviors.

Behavior change theories are also used for understanding behaviors related to treatment adherence. Understanding the factors that hinder or promote health behaviors is central to the transtheoretical model [29], which we used to guide the incorporation of patient-centered, tailored information and feedback as the intervention focused heavily on the initiation and maintenance phases of behavior change [29]. While a generic health care–administered intervention may improve treatment adherence through reminders, a tailored intervention can address issues that are specifically relevant to each patient.

Thus, drawing on the stages of change [29] and the revised health decision model [28,29], the STOP DKD APP intervention helped patients to (1) set healthy goals and gain self-efficacy, (2) implement healthy behaviors and monitor performance, and (3) maintain the behaviors and associated risk factor control over time [35,36]. Together, these models directed the structure, content, and format of the intervention in order to optimize medical management of this population while encouraging patient engagement in and adherence to their self-management behaviors.

The telephone-based intervention was administered by a call center using nonclinician case managers. The intervention consisted of (1) monthly telephone calls for 6 months, (2) monthly emails that summarized the content covered with the case manager during the monthly phone call, and (3) weekly text messages. Table 1 provides examples of the content in the text messages and monthly calls. Patients received medication adherence information and e-reminders to take DKD-related medications and self-monitor their health status (eg, SBP, weight). This study did not include medical management for hypertension. All intervention components were designed to be culturally sensitive, and content addressed self-management facilitators and barriers common in this population. Additionally, all content provided was tailored to patient responses about self-management behaviors, medication adherence, smoking status, and prescribed medications. Patient responses were obtained during interactions with the case manager or the interactive text messages received during the intervention. All content was presented at a literacy level below a 6th grade reading level.

The monthly call was a review of education and self-management topics related to diabetes, hypertension, and hyperlipidemia. During these calls, the case manager reviewed the patient’s current medications, changes in medication status, or side effects the patient was experiencing and then provided self-management content based on the patient’s self-reported health status. Phone calls provided patients the opportunity to obtain personalized, disease-specific information and feedback. If a patient could not be reached on schedule, the case manager attempted to complete the phone call at the next scheduled call time. Once a call was completed, the call schedule was adjusted accordingly. Following the completion of each monthly phone call, patients were sent an email that summarized the content discussed in the phone call.

Patients received text messages containing information related to self-management and medication adherence on diabetes and hypertension, including messages on side effects, risks and benefits of medication treatment, and barriers to medication adherence. These text messages prompted patients to engage in self-management behaviors (“Remember to carry a snack or a source of sugar with you in case your blood sugar gets low”), provided education (“Exercise lowers your risk for heart disease and stroke, relieves stress, and strengthens your heart, muscles, and bones”), and offered suggestions (“You can decrease your salt intake by cutting back on fast foods and processed foods such as canned soups and vegetables and frozen dinners” and “To help prevent low blood sugar, eat your meals and snacks at the same time each day. Do not skip meals”). Text messages were sent 3 times each week around 6 pm for 6 months.

### Measures

The focus of the intervention was to optimize medication management related to treatment for diabetes and hypertension. The primary study outcome was change in SBP, operationalized as controlled (SBP <140 mm Hg) or poorly controlled (SBP ≥140 mm Hg) from baseline to 90 days after the last completed phone call. Baseline SBP was defined as the SBP closest to study enrollment within a window from 1 year prior to 14 days into the study period. Completion SBP was defined as the closest SBP measurement to 90 days after the last completed phone call from a window spanning last call day to 180 days later. Our selection of these intervals represents our pragmatic approach to address potential sparsity of data for some individuals. Engagement was recorded as the number of completed monthly calls at the time of each SBP measurement. Individuals who completed 4 or more calls were considered engaged during the study period for pre-post comparisons, while those completing fewer were considered nonengaged.

### Analyses

We examined engagement in the intervention to determine feasibility of providing an mHealth intervention to patients from an FQHC. We defined level of engagement as the number of completed phone calls during the intervention.

We used two statistical methods to evaluate the effectiveness of the STOP-DKD APP intervention. First, we used the McNemar test [37,38] to compare the proportion of patients who went from having a controlled blood pressure (SBP <140 mm Hg) or poorly controlled pressure (SBP ≥140 mm Hg) at baseline to the opposite category at the end of the study period. Second, using linear regression, we compared change in SBP over the time-in-study of those patients who had a controlled SBP at baseline to those patients who had a high SBP at baseline, adjusting for the level of study engagement and its interaction with baseline SBP. This approach allowed testing for a change over time, whether greater participation in the intervention was associated with a greater impact on SBP, and if that impact was different for those with high versus controlled SBP at baseline.

**Table 1:** Description of case manager call and text message topics.

Month	Phone calls	Text messages
1	Review of medications and side effects	Weekly medication reminders and study introduction
	Hypertension and cardiovascular disease knowledge	Stress and increased blood pressure
2	Review of medications and side effects	Weekly medication reminders
	Hypoglycemia and foot care	Hyperglycemia recognition
	Diabetes medications and side effects	Tobacco use
3	Review of medications and side effects	Weekly medication reminders
	Hyperlipidemia medications and side effects	Alcohol
	Diet and weight	Sleep health
	Hyperlipidemia knowledge	Cholesterol and blood pressure knowledge
4	Review of medications and side effects	Weekly medication reminders
	Hypertension medications	Patient and provider or clinic communication
	Exercise	Blood pressure and diabetes knowledge
	Sleep	Diet (lowering carbohydrates)
5	Review of medications and side effects	Weekly medication reminders
	Depression	Diet (carbohydrates and fiber)
	Tobacco use	Cholesterol knowledge
6	Review of medications, side effects, and aspirin use	Weekly medication reminders
	Alcohol knowledge	Blood pressure and blood sugar goals
	Patient-provider interaction	Patient and provider or clinic communication

## Results

### Sample Characteristics

**Figure 1:**
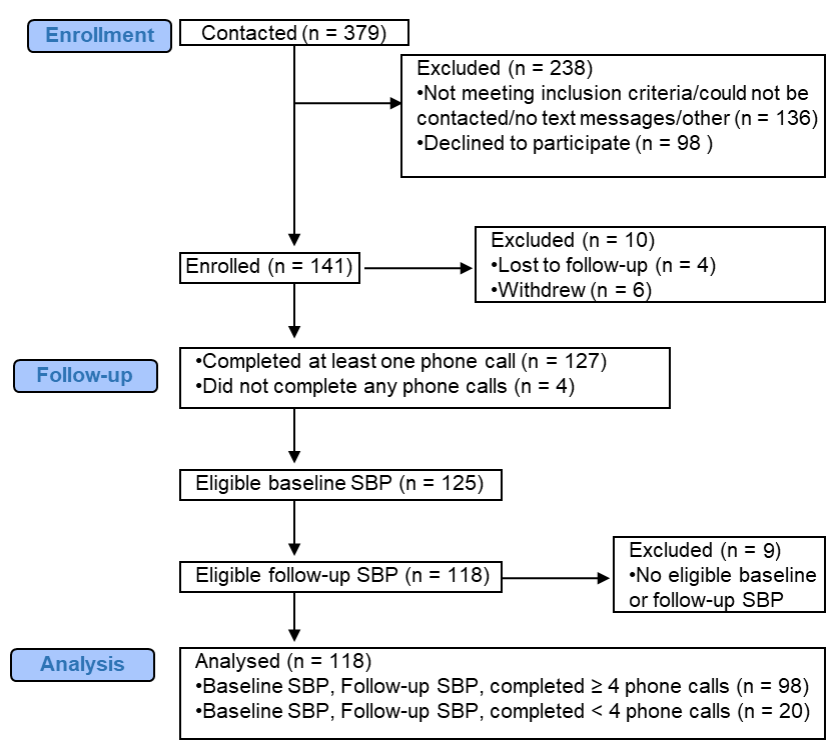
Consolidated Standards of Reporting Trials diagram.

We contacted 379 patients about the STOP DKD APP study; of those patients, we excluded 238. We enrolled 141 patients from May 2015 through January 2016 (see Figure 1 for the Consolidated Standards of Reporting Trials diagram). In total, 127 patients completed at least one phone call. Of those 127 patients, 125 had a baseline SBP and 118 had both a baseline and follow-up SBP. Therefore, the following analyses focused on the 118 patients (118/141, 83.7%) who completed 1 or more phone call and had both a baseline and follow-up SBP. These analyses do not include patients who withdrew from the study (6/141, 4.3%), were lost to follow-up (4/141, 2.8%), did not complete any phone calls (4/141, 2.8%), or did not have an eligible baseline or follow-up SBP (9/141, 6.4%).

Among the 118 individuals, the mean age was 56.9 years. Patients were primarily female (73/118, 61.9%), self-identified as nonwhite or black (103/118, 87.3%), and had a high school education or more (91/118, 77.1%). Most had no insurance (35/118, 29.7%) or government-funded support or special programs (53/118, 44.9%), while 25.4% (30/118) had private/commercial insurance. In regard to baseline clinical characteristics, the mean 12-month prior SBP of the sample was 139.5 (SD 19.8) mm Hg and mean DBP was 82.5 (SD 11.2) mm Hg and most had an estimated glomerular filtration rate (eGFR) >60 (74/118, 62.7%). The sample is fully described in Table 2.

**Table 2:** Baseline demographic and clinical characteristics (n=118).

Characteristic		Value
Age in years, mean (SD)		56.9 (7.6)
Gender, female, n (%)		73 (61.9)
**Race, n (%)**		
	White	15 (12.7)
	Black	99 (83.9)
	Other	4 (3.4)
**Ethnicity (Hispanic) , n (%)**		
	Yes	2 (1.7)
	No	114 (96.6)
	Refused	2 (1.7)
	Marital status, married, n (%)	25 (21.2)
	Missed medication, yes, n (%)	57 (48.3)
**Insurance status, n (%)**		
	Self-pay	35 (29.7)
	Commercial	30 (25.4)
	Government or special program	53 (44.9)
**Access to computer, n (%)**		
	Yes	54 (45.8)
	No	63 (53.4)
	Missing	1 (0.8)
	Education, high school graduate or greater, n (%)	91 (77.1)
	Ability to do usual activities, no problems reported, n (%)	65 (55.1)
	Ability to take care of self, no problems washing or dressing, n (%)	101 (85.6)
	Self-rated health at baseline (0 to 100), mean (SD)	65.6 (23.6)
	Systolic blood pressure (mm Hg), mean (SD)	139.5 (19.8)
	Diastolic blood pressure (mm Hg), mean (SD)	82.5 (11.2)
**Estimated glomerular filtration rate, n (%)**		
	<60	24 (20.3)
	>60	74 (62.7)
	Missing	20 (16.9)

**Table 3:** Baseline demographic and clinical characteristics by engagement.

Characteristic		Nonengaged (n=20)	Engaged (n=98)
Age in years, mean (SD)		50.3 (8.8)	58.2 (6.6)
Gender, female, n (%)		16 (80)	57 (58)
**Race, n (%)**			
	White	3 (15)	12 (12)
	Black	17 (85)	82 (84)
	Other	0 (0)	4 (4)
**Ethnicity (Hispanic), n (%)**			
	Yes	0 (0)	2 (2)
	No	20 (100)	94 (96)
	Refused	0 (0)	2 (2)
	Marital status, married, n (%)	5 (25)	20 (20)
	Missed medication, yes, n (%)	10 (50)	47 (48)
**Insurance status, n (%)**			
	Self-pay	5 (25)	30 (31)
	Commercial	4 (20)	26 (27)
	Government or special program	11 (55)	42 (43)
**Access to computer, n (%)**			
	Yes	9 (45)	45 (46)
	No	11 (55)	52 (53)
	Missing	0 (0)	1 (1)
	Education, high school graduate or greater, n (%)	17 (85)	74 (76)
	Ability to do usual activities, no problems reported, n (%)	8 (40)	57 (58)
	Ability to take care of self, no problems washing or dressing, n (%)	18 (90)	83 (85)
	Self-rated health at baseline (0-100), mean (SD)	70.1 (23.7)	64.6 (23.5)
	Systolic blood pressure (mm Hg), mean (SD)	138.2 (21.9)	139.8 (19.5)
	Diastolic blood pressure (mm Hg), mean (SD)	85.1 (7.6)	82.0 (11.8)
**Estimated glomerular filtration rate, n (%)**			
	<60	3 (15)	21 (21)
	>60	13 (65)	61 (62)
	Missing	4 (20)	16 (16)

### Engagement

We noted a few differences between engaged and nonengaged patients. Engaged patients (completed 4 or more phone calls) had a significantly higher mean age (*P*=.001, Wilcoxon rank-sum test) and were marginally more likely to be male (*P*=.08, Fisher exact test) and have a diagnosis of hypertension than nonengaged patients (*P*=.09, Fisher exact test). The baseline demographic and clinical characteristics by study engagement over the course of the intervention are presented in Table 3, and other than those reported above, no other differences were seen between engaged and nonengaged participants.

**Table 4:** Pre-post change in systolic blood pressure control status.

Baseline SBP	Postintervention SBP^a^		
	Control	Poor control	Total
Control	46 (39.0)	22 (18.6)	68
Poor control	19 (16.1)	31 (26.3)	50
Total	65	53	118

^a^SBP: systolic blood pressure.

### Clinical Outcomes

The proportion of patients with poorly controlled SBP at the start of the study (50/118, 42.4%) did not change significantly at study completion (53/118, 44.9%) (*P*=.64). The proportions who switched from in control (defined as SBP <140 mm Hg) to poor control (defined as SBP ≥140 mm Hg) (22/118, 18.6%) and poor control to in control (19/118, 16.1%) were comparable (*P*=.64, McNemar test) (Table 4).

The McNemar test does not consider the magnitude of changes over time, and slight changes could reclassify a patient into the other group. Thus, we compared the rates of change in SBP while accounting for baseline SBP and engagement with the study. Participants who completed 4 or more phone calls (98/118, 83.1%) did not experience a statistically significant decrease in SBP when compared to those who completed fewer calls.

## Discussion

### Principal Findings

This study is among the first to examine the use of mHealth supported with phone calls from nonclinician case managers in a predominately minority and low-income sample. We successfully recruited and engaged a sample of patients with diabetes and poorly controlled hypertension from an FQHC for a multifactorial behavioral-educational population intervention primarily using the EHR. Individuals who completed 4 or more phone calls (out of 6) did not experience a statistical decrease in SBP relative to those patients who were not as engaged. Overall, the findings provide insight into designing population health management programs that aim to address modifiable risk factors for patients with both diabetes and poorly controlled hypertension.

The identification of patients using a system linked to an EHR can quickly detect patients at high risk for poor diabetes outcomes. EHRs can facilitate population health management because they facilitate the collection of patient data on a large scale and enable a more rapid and efficient analysis of these patient data [39]. The early identification of patients at risk for poor health outcomes, such as those with diabetes and poorly controlled hypertension, can decrease the progression of renal and cardiovascular complications and potentially decrease disparities in treatment and care [40]. The STOP-DKD APP study successfully facilitated the identification of patients at risk for DKD via a community clinic’s EHR and provided education tailored to each patient.

Our study successfully recruited a black sample with limited financial resources from an FQHC for an mHealth intervention. The sample was highly engaged, with the majority completing 4 or more case manager–administered, telephone-delivered self-management education calls. One potential reason for high engagement could be the bidirectional monthly interaction with a case manager. These frequent encounters with the case manager may have addressed the patient’s current questions about self-management and medication adherence in a more timely fashion than an episodic appointment with a medical provider [41,42]. This study’s pragmatic design was an additional strength as there was no in-person contact, the intervention was administered by telephone, and blood pressure was assessed via each patient’s EHR. Taken together, these strengths indicate that mHealth interventions in which routine encounters are delivered by nonclinicians can be used with high-risk, low-income populations without placing undue burden on clinicians at community health clinics.

There are several potential explanations for why this multifactorial intervention was not as impactful as hypothesized. First, our sample’s blood pressure was relatively well controlled at baseline, which may have limited our ability to detect the clinical impact of improved SBP. Second, the intervention’s dose of self-management education and/or the length of the intervention may not have been sufficient to affect cardiovascular disease risk factor control in this low-income population. Additionally, clinic staff may have given more attention to patients at baseline who had poorly controlled hypertension and may have focused less on those patients who had controlled blood pressure at baseline. Collectively, our findings reflect the literature that further research is needed on pragmatic, multifactorial interventions that address chronic illness self-management [43]. A greater understanding of this population of low-income individuals with chronic illness will help identify the optimal intervention dose and length, intervention strategies, and message content to impact metabolic outcomes.

Another reason for the limited impact on blood pressure may have been due to the use of routine clinic measurements for outcome ascertainment. The variance in frequency of blood pressure values for each patient may have impacted our results due to the imprecise nature of voluntary patient visits to the FQHC. For example, to obtain the SBP and DBP from the FQHC’s EHR, we aimed to get as close as possible to a 3-month follow-up window during the 6-month study period. As a result, this limited our ability to collect data that was in sync with the completed phone calls. The SBP measurements used in this study for each individual were a result of the patients voluntarily going to the FQHC and having a blood pressure value logged into the system during the intervention time period. Additionally, the precision of the blood pressure readings may have been low, as only one blood pressure reading may have been taken during each clinic visit [44]. Variability in routine clinic blood pressure measurement has been increasingly recognized [45,46], so although university-affiliated clinics have standard procedures for BP measurement, it is possible that routine clinic practice and all available clinic data may have introduced error into our analysis. Our experience highlights the challenges of using clinical measures for outcomes and indicates the need for further research on pragmatic methods to accurately obtain clinical outcome data from patients who receive care at an FQHC.

### Limitations

There are several limitations to this study. First, we sampled individuals who received medical care at an FQHC, which may limit generalizability of these results. Second, the patients in this study had a mean SBP of 140 mm Hg at baseline. The wide variance in the mean SBP may be because we included patients with any SBP over 140 mm Hg, which may have led to a mean SBP at baseline near the target threshold. Third, potential reasons for the minimal results seen with this study could be because of limited data in the EHR due to patients’ variable clinic visits and the lack of standardization of the blood pressure measurement. In addition, the study’s inclusion criteria may not have been stringent enough to identify patients with poorly controlled hypertension who may have benefited from inclusion in this intervention. However, despite these limitations we believe the findings from this study add to the literature on engagement in mHealth interventions among patients who receive care at an FQHC.

### Conclusions

The findings from this study indicate that the combination of an automated system that identifies at-risk patients using an EHR in addition to tailored education via mHealth can successfully be used to provide self-management education to a high-risk, low-income population. The findings from this study indicate that population health applications can be easily applied with a potentially promising impact in an FQHC.

## Abbreviations

BP: blood pressure
DBP: diastolic blood pressure
DKD: diabetic kidney disease
EHR: electronic health record
FQHC: federally qualified health center
ICD: International Classification of Diseases
mHealth: mobile health
SBP: systolic blood pressure
STOP-DKD APP: Simultaneous Risk Factor Control Using Telehealth to Slow Progression of Diabetic Kidney Disease Automated Population Program
VA: Veterans Affairs
